# Gold nanoparticles improve motility, functional integrity, and antioxidant status of boar spermatozoa during liquid storage

**DOI:** 10.3389/fvets.2026.1909055

**Published:** 2026-07-30

**Authors:** Dipan Rudra Paul, Sourabh Deori, Himsikha Chakravarty, Rahul Katiyar, Mahak Singh, Sandeep Ghatak, Govindasamy Kadirvel

**Affiliations:** 1 ICAR Research Complex for NEH Region, Umiam, Meghalaya, India; 2 ICAR-Indian Veterinary Research Institute, Bareilly, Uttar Pradesh, India; 3 ICAR Research Complex for NEH Region, Nagaland Centre, Medziphema, Nagaland, India

**Keywords:** boar semen, BTS extender, gold nanoparticles, liquid storage, oxidative stress

## Abstract

Liquid preservation of boar semen is widely used in artificial insemination programs; however, storage-induced oxidative stress progressively impairs sperm function and reduces fertility potential. The present study evaluated the effects of gold nanoparticles (AuNPs) on sperm quality, functional integrity, and oxidative status of liquid-preserved boar semen during storage at 17 °C. 24 ejaculates collected from four healthy Lumsniang breeding boars were diluted in Beltsville Thawing Solution (BTS) extender and allocated to three treatment groups: T0 (control, no AuNPs), T1 (5 million AuNPs/ml), and T2 (10 million AuNPs/ml). Extended semen samples were stored for 5 days and evaluated on days 0, 3, and 5. Sperm motility and kinematic parameters were assessed using computer-assisted semen analysis (CASA), while viability, plasma membrane integrity, acrosomal integrity, and mitochondrial membrane potential were determined using CFDA/PI staining, hypo-osmotic swelling test, FITC-PNA staining, and JC-1 staining, respectively. Oxidative status was assessed by measuring malondialdehyde (MDA) concentration and total antioxidant capacity (TAC). Storage duration significantly affected all sperm quality parameters (*P* < 0.05), resulting in progressive declines in motility, viability, membrane and acrosomal integrity, mitochondrial activity, and antioxidant capacity, accompanied by increased lipid peroxidation. However, AuNP supplementation significantly improved semen preservation compared with the control group. On day 5, semen supplemented with 10 million AuNPs/ml (T2) exhibited higher total motility (30.78 ± 1.53%), progressive motility (17.97 ± 0.94%), viability (47.62 ± 1.38%), plasma membrane integrity (17.25 ± 0.44%), acrosomal integrity (54.91 ± 0.96%), and high mitochondrial membrane potential (41.79 ± 0.79%) than the control (*P* < 0.05). Furthermore, T2 showed lower MDA concentrations and higher TAC values, indicating enhanced antioxidant protection during storage. These findings demonstrate that AuNP supplementation, particularly at 10 million AuNPs/ml, effectively mitigates oxidative stress and preserves sperm functionality during liquid storage, suggesting its potential application for improving the shelf life and quality of boar semen used in artificial insemination programs.

## 1 Introduction

In porcine artificial insemination, the use of frozen–thawed semen is often associated with reduced fertility outcomes and smaller litter sizes; consequently, liquid-preserved semen is presently the preferred choice for AI programs (1). Liquid semen kept at 15–18 °C for up to 5 days is used in over 99% of inseminations globally (2, 3). However, during storage, sperm quality gradually declines, resulting in decreased fertilizing ability and reproductive efficiency (4). Because of the large concentration of polyunsaturated fatty acids (PUFAs) in their plasma membrane as well as the comparatively low antioxidant capacity of seminal plasma, boar spermatozoa are extremely vulnerable to oxidative stress (5). Reactive oxygen species (ROS) are continuously produced by normal sperm metabolism during liquid storage, especially at the mitochondrial along with plasma membrane levels (6). Excessive ROS generation upsets the cellular redox equilibrium and causes oxidative stress, even though physiological quantities of ROS are necessary for sperm capacitation, acrosome reaction, and sperm-oocyte contact (7). Overproduction of ROS causes membrane PUFAs to undergo lipid peroxidation, which leads to membrane integrity loss, reduced mitochondrial activity, reduced motility and viability, acrosomal damage and DNA breakage (8). Additionally, oxidative stress is thought to be one of the main causes of the deterioration in quality of sperm during boar semen liquid preservation. As a result, adding antioxidants to semen extenders has become a viable method for reducing oxidative damage and extending the life of sperm during storage.

Gold nanoparticles (AuNPs) have attracted considerable interest among metallic nanoparticles owing to their remarkable physicochemical stability, excellent biocompatibility, and promising antioxidant properties, making them highly suitable for biomedical and reproductive applications (9). Several studies have demonstrated that supplementation with gold nanoparticles (AuNPs) can improve sperm motility, membrane integrity, and overall sperm quality while reducing oxidative damage during semen storage and cryopreservation, thereby enhancing sperm preservation outcomes (10, 11). Supplementation of gold nanoparticles to goat semen extender enhanced sperm motility, viability, membrane integrity, acrosomal integrity, and antioxidant status after cryopreservation (12). Similarly, buffalo semen augmented with metallic nanoparticles, such as AuNPs, showed better fertility, decreased oxidative stress, and improved post-thaw sperm quality (11). Antimicrobial agents are commonly added into semen extenders to inhibit bacterial and fungal contamination during storage (13, 14); however, gold nanoparticles (AuNPs), owing to their broad-spectrum antimicrobial properties have emerged as a promising alternative or complementary additive for improving semen preservation while reducing reliance on conventional antibiotics. Information on the application of gold nanoparticles (AuNPs) in liquid-preserved boar semen is scarce. Therefore, this study investigated the effects of AuNPs supplementation in Beltsville Thawing Solution (BTS) extender on sperm quality, functional integrity, and oxidative status during short-term storage of boar semen at 17 °C.

## 2 Materials and methods

### 2.1 Ethical approval

All experimental procedures involving animals were approved by the Institutional Animal Ethics Committee (IAEC) of ICAR Research Complex for NEH Region, Umiam, Meghalaya, India (Approval No. 2100/GO/RBi/L/2020/CCSEA) and were conducted in accordance with the guidelines of the Committee for Control and Supervision of Experiments on Animals (CCSEA), Government of India.

### 2.2 Experimental animals and semen collection

The study was conducted using four healthy Lumsniang breeding boars (75% Hampshire × 25% Niang Megha) aged between 18 and 24 months and maintained under uniform feeding and management conditions at the Livestock Farm, ICAR Research Complex for NEH Region, Umiam, Meghalaya, India. A total of 24 ejaculates (six ejaculates per boar) were collected twice weekly using the gloved-hand technique with the aid of a dummy sow.

Immediately after collection, semen samples were transported to the laboratory in a thermos flask and maintained at 37 °C in a water bath until evaluation. Only ejaculates meeting the following quality criteria were included in the study: gel-free semen volume of 100–250 ml, progressive motility ≥70%, and sperm concentration ≥200 × 106 sperm/ml.

### 2.3 Semen processing and experimental design

The selected ejaculates were diluted in Beltsville Thawing Solution (BTS) extender and divided into three equal aliquots. The first aliquot served as the control group (T0) without supplementation. The second (T1) and third (T2) aliquots were supplemented with 20 nm gold nanoparticles (AuNPs; Product No. 753610, Sigma-Aldrich, USA) at concentrations of 5 million AuNPs/ml and 10 million AuNPs/ml, respectively.

The extended semen samples were stored at 17 °C in a biological oxygen demand (BOD) incubator for up to 5 days. Semen quality assessments were performed on days 0, 3, and 5 of storage.

### 2.4 Evaluation of sperm motility and kinematic parameters

Sperm motility and kinematic characteristics were evaluated using a computer-assisted semen analysis (CASA) system (Biovis CASA, Expert Vision Labs Pvt. Ltd., India) with a Leja 20 μm counting chamber (Leja Products B.V., Nieuw-Vennep, The Netherlands) following the method described by Jawad et al. (15). Briefly, 2 μl of diluted semen was loaded into a pre-warmed counting chamber and analyzed at room temperature. At least 500 spermatozoa from five randomly selected microscopic fields were evaluated per sample. The CASA system operated at a frame rate of 30 frames/s.

The parameters recorded included total motility (TMOT, %), progressive motility (PMOT, %), curvilinear velocity (VCL, μm/s), average path velocity (VAP, μm/s), straight-line velocity (VSL, μm/s), linearity (LIN, %), straightness (STR, %), wobble (WOB, %), and beat-cross frequency (BCF, Hz). Spermatozoa with VSL >25 μm/s and STR ≥75% were classified as progressively motile.

### 2.5 Assessment of sperm viability

Sperm viability was assessed using carboxyfluorescein diacetate and propidium iodide (CFDA/PI) dual fluorescent staining according to Kukov et al. (16) with minor modifications. Briefly, 200 μl of diluted semen was mixed with 3 μl CFDA (0.46 mg/ml in dimethyl sulfoxide) and 4 μl PI (0.5 mg/ml in phosphate-buffered saline; PBS). The samples were incubated at 37 °C for 30 min in the dark. Following incubation, smears were prepared on clean glass slides and examined under a fluorescence microscope (Nikon Eclipse 80i, Nikon Corporation, Japan) using excitation wavelengths of 465–495 nm and 540 nm. A minimum of 200 spermatozoa were evaluated per sample.

### 2.6 Assessment of plasma membrane integrity

Functional integrity of the sperm plasma membrane was evaluated using the hypo-osmotic swelling test (HOST) following the procedure of Jeyendran et al. (17). Semen samples were incubated in hypo-osmotic solution at 37 °C and examined under a phase-contrast microscope. Spermatozoa exhibiting characteristic tail swelling were considered membrane-intact.

### 2.7 Assessment of acrosomal integrity

Acrosomal integrity was assessed using fluorescein isothiocyanate-conjugated peanut agglutinin (FITC-PNA) staining as described by Esteves et al. (18) with slight modifications. Briefly, 20 μl of semen was mixed with 100 μl PBS and incubated at 37 °C for 30 min. Smears were prepared on clean glass slides, air-dried, and examined under a fluorescence microscope (Nikon Eclipse 80i, Japan) equipped with a FITC filter (excitation wavelength 490 nm). At least 200 spermatozoa were evaluated per sample.

### 2.8 Assessment of mitochondrial membrane potential

Mitochondrial membrane potential (MMP) was determined using JC-1 fluorescent dye following the protocol described by Katiyar et al. (19). Briefly, 100 μl of diluted semen was mixed with 1 μl JC-1 dye and 4 μl PI and incubated at 37 °C for 30 min in the dark. Following incubation, smears were prepared and examined under a fluorescence microscope (Nikon Eclipse 80i, Japan) using an excitation wavelength of 540 nm. Spermatozoa exhibiting orange-red fluorescence were considered to possess high mitochondrial membrane potential.

### 2.9 Determination of oxidative stress markers

#### 2.9.1 Malondialdehyde (MDA)

Lipid peroxidation was assessed by measuring malondialdehyde (MDA) concentration using the thiobarbituric acid reactive substances (TBARS) assay according to Suleiman et al. (20) with minor modifications. Semen samples were reacted with thiobarbituric acid-trichloroacetic acid (TBA-TCA) reagent and heated in a boiling water bath. After centrifugation, absorbance of the supernatant was measured at 535 nm using a UV–visible spectrophotometer. MDA concentration was calculated using a molar extinction coefficient of 1.56 × 10^5^ mol^−1^ cm^−1^ and expressed as μmol/ml.

#### 2.9.2 Total antioxidant capacity (TAC)

Total antioxidant capacity (TAC) was determined using a commercial colorimetric assay kit (ABTS Enzyme Method; Thermo Fisher Scientific, Catalog No. EEA023) according to the manufacturer's instructions. Results were expressed as nmol/ml.

### 2.10 Statistical analysis

Data were analyzed using IBM SPSS Statistics version 27.0 (IBM Corp., Armonk, NY, USA). Results are presented as mean ± standard error of the mean (SEM). The effects of treatment, storage duration, and their interaction were evaluated using two-way analysis of variance (ANOVA). When significant differences were detected, means were compared using Duncan's multiple range test. Differences were considered statistically significant at *P* < 0.05.

## 3 Results

### 3.1 Effect of AuNP supplementation on CASA motion characteristics during liquid storage

The effects of gold nanoparticle (AuNP) supplementation on CASA-derived sperm motion characteristics during liquid storage are presented in Table 1. At semen dilution (day 0), total motility (TMOT) did not differ significantly among treatments and ranged from 87.69 ± 1.07% to 88.42 ± 1.05%. A significant decline (*P* < 0.01) in TMOT was observed in all groups during storage; however, the reduction was less pronounced in AuNP-supplemented treatments. On day 3, TMOT was significantly higher in T2 (59.59 ± 1.95%) and T1 (54.40 ± 1.99%) compared with T0 (48.73 ± 1.81%). By day 5, T2 maintained the highest TMOT (30.78 ± 1.53%), followed by T1 (27.30 ± 1.57%) and T0 (25.11 ± 1.18%).

**Table 1:** Effect of different doses of AuNPs supplementation on semen extender in CASA parameters.

Parameters	Day	T0	T1	T2	*P*-value
TMOT (%)	0	87.69 ± 1.07^A^	87.85 ± 1.02^A^	88.42 ± 1.05^A^	0.876^NS^
	3	48.73 ± 1.81^bB^	54.40 ± 1.99^aB^	59.59 ± 1.95^aB^	0.001^**^
	5	25.11 ± 1.18^bC^	27.30 ± 1.57^abC^	30.78 ± 1.53^aC^	0.024^*^
	*P*-value	0.000^**^	0.000^**^	0.000^**^	
PMOT (%)	0	76.88 ± 1.09^A^	77.49 ± 1.22^A^	78.02 ± 1.16^A^	0.788^NS^
	3	35.22 ± 2.06^bB^	41.44 ± 2.34^abB^	46.93 ± 2.30^aB^	0.002^**^
	5	11.31 ± 0.37^cC^	13.61 ± 0.58^bC^	17.97 ± 0.94^aC^	0.000^**^
	*P*-value	0.000^**^	0.000^**^	0.000^**^	–
VCL (μm/s)	0	120.61 ± 5.24^A^	122.13 ± 5.83^A^	127.74 ± 5.81^A^	0.643^NS^
	3	83.24 ± 0.86^bB^	84.88 ± 1.47^bB^	92.31 ± 2.15^aB^	0.000^**^
	5	72.97 ± 0.75^bC^	78.98 ± 1.47^aB^	79.58 ± 1.34^aC^	0.000^**^
	*P*-value	0.000^**^	0.000^**^	0.000^**^	–
VAP (μm/s)	0	60.78 ± 2.21^bA^	63.33 ± 2.49^bA^	72.38 ± 3.11^aA^	0.007^**^
	3	33.17 ± 0.38^cB^	38.82 ± 0.60^bB^	42.99 ± 0.80^aB^	0.000^**^
	5	24.63 ± 0.76^bC^	33.32 ± 0.57^aC^	34.45 ± 0.53^aC^	0.000^**^
	*P*-value	0.000^**^	0.000^**^	0.000^**^	–
VSL (μm/s)	0	54.55 ± 1.91^bA^	58.30 ± 2.31^bA^	67.50 ± 2.91^aA^	0.001^**^
	3	24.78 ± 0.28^cB^	33.13 ± 0.68^bB^	37.77 ± 0.65^aB^	0.000^**^
	5	18.42 ± 1.10^bC^	27.11 ± 0.71^aC^	28.46 ± 0.84^aC^	0.000^**^
	*P*-value	0.000^**^	0.000^**^	0.000^**^	–
LIN (%)	0	45.70 ± 1.12^cA^	48.43 ± 0.81^bA^	53.87 ±± 0.64^aA^	0.000^**^
	3	30.15 ± 0.88^bB^	40.62 ± 1.56^aB^	42.42 ± 0.76^aB^	0.000^**^
	5	26.66 ± 2.06^bB^	37.41 ± 1.21^aB^	39.36 ± 1.65^aB^	0.000^**^
	*P*-value	0.000^**^	0.000^**^	0.000^**^	–
STR (%)	0	87.33 ± 0.40^cA^	90.68 ± 0.42^bA^	92.56 ± 0.26^aA^	0.000^**^
	3	69.63 ± 1.63^cB^	81.04 ± 1.04^bB^	87.00 ± 0.47^aB^	0.000^**^
	5	64.93 ± 2.46^bB^	74.44 ± 1.78^aC^	75.29 ± 1.91^aC^	0.001^**^
	*P*-value	0.000^**^	0.000^**^	0.000^**^	–
WOB (%)	0	51.05 ± 1.04^bA^	52.71 ± 0.87^bA^	57.65 ± 0.61^aA^	0.000^**^
	3	40.44 ± 0.31^bB^	47.41 ± 1.34^aB^	48.09 ± 0.54^aB^	0.000^**^
	5	35.89 ± 1.47^bC^	45.49 ± 0.67^aB^	47.37 ± 1.08^aB^	0.000^**^
	*P*-value	0.000^**^	0.000^**^	0.000^**^	–
BCF (Hz)	0	24.45 ± 0.65^bA^	25.56 ± 0.72^bA^	27.90 ± 0.84^aA^	0.006^**^
	3	14.63 ± 0.12^cB^	18.07 ± 0.12^bB^	20.10 ± 0.37^aB^	0.000^**^
	5	10.73 ± 0.55^bC^	15.07 ± 0.27^aC^	15.73 ± 0.29^aC^	0.000^**^
	*P*-value	0.000^**^	0.000^**^	0.000^**^	–

^**^p < 0.01; ^*^p < 0.05; ^NS^Non-significant.

Means with different superscripts in a row (a, b, c) differed significantly.

Means with different superscripts in a column (A, B, C) differed significantly.

Progressive motility (PMOT) exhibited a similar trend. Initial values ranged from 76.88 ± 1.09% to 78.02 ± 1.16%, with no treatment differences. By day 5, PMOT declined significantly in all groups but remained higher in T2 (17.97 ± 0.94%) compared with T1 (13.61 ± 0.58%) and T0 (11.31 ± 0.37%) (*P* < 0.01).

Among velocity parameters, curvilinear velocity (VCL), average path velocity (VAP), and straight-line velocity (VSL) were significantly higher in AuNP-treated groups during storage. On day 5, T2 recorded the highest VCL (79.58 ± 1.34 μm/s), VAP (34.45 ± 0.53 μm/s), and VSL (28.46 ± 0.84 μm/s). Similarly, linearity (LIN), straightness (STR), wobble (WOB), and beat-cross frequency (BCF) were significantly improved in AuNP-supplemented semen. On day 5, LIN values were 26.66 ± 2.06%, 37.41 ± 1.21%, and 39.36 ± 1.65% in T0, T1, and T2, respectively, while corresponding STR values were 64.93 ± 2.46%, 74.44 ± 1.78%, and 75.29 ± 1.91%. BCF was highest in T2 (15.73 ± 0.29 Hz) and lowest in T0 (10.73 ± 0.55 Hz) (*P* < 0.01).

### 3.2 Effect of AuNP supplementation on sperm functional attributes

**Figure 1:**
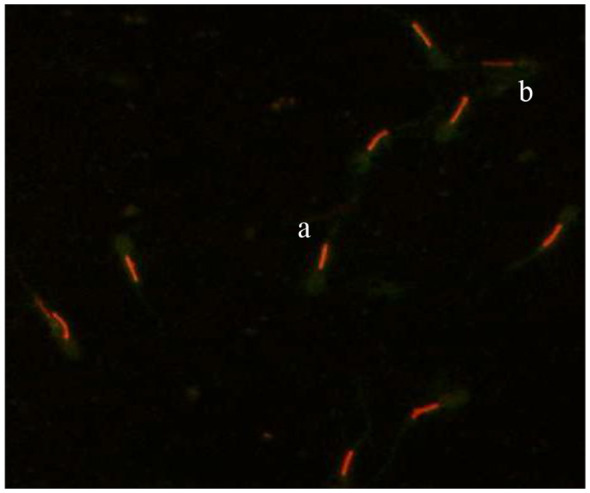
Mitochondrial membrane potential by JC-1 stain (a: High MMP; b: Low MMP).

**Figure 2:**
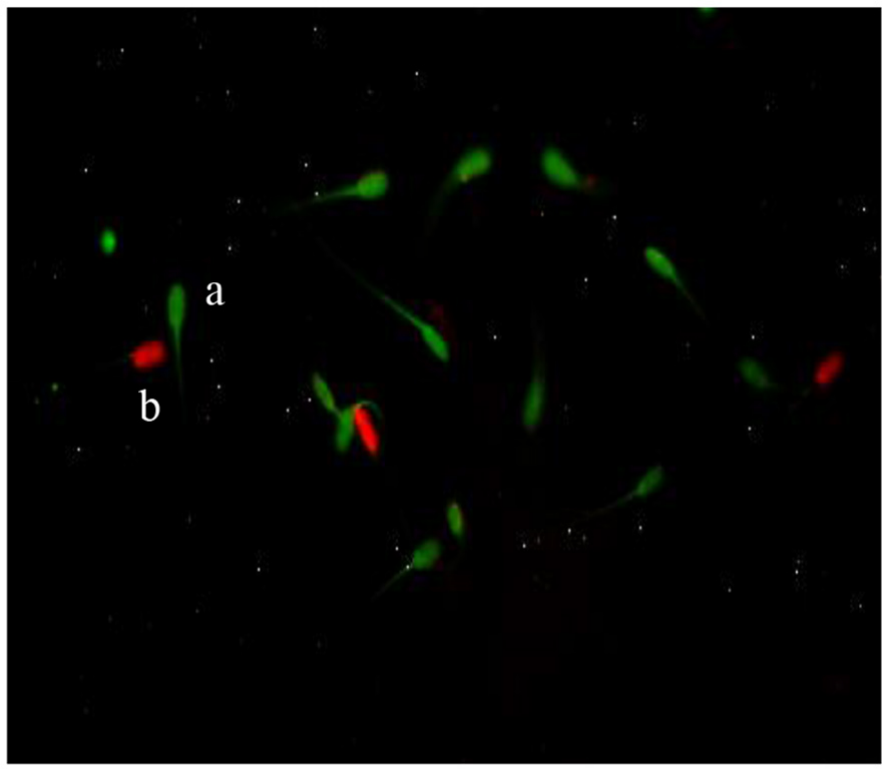
Sperm viability by CFDA and PI stain (a: Live; b: Dead).

**Figure 3:**
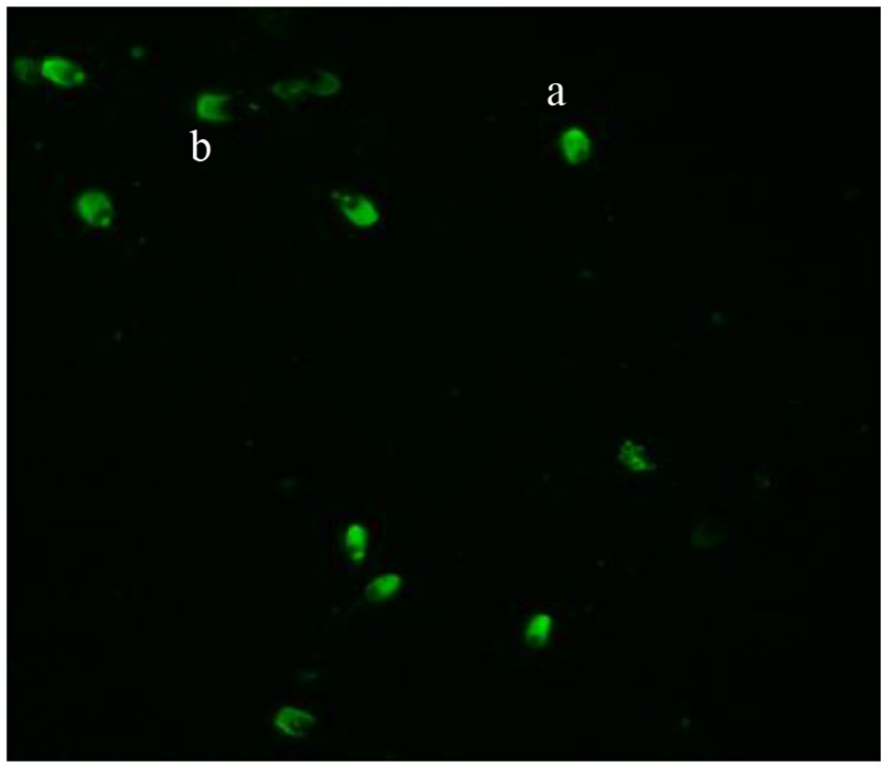
Acrosomal integrity by FITC-PNA (a: Intact acrosome; b: Damaged acrosome).

The effects of AuNP supplementation on sperm viability, plasma membrane integrity, acrosomal integrity, and mitochondrial membrane potential (MMP) are presented in Table 2 and Figures 1–3.

**Table 2:** Effect of different doses of AuNPs supplementation on semen extender in various seminal attributes.

Parameters	Day	T0	T1	T2	*P*-value
Viability (%)	0	89.62 ± 0.40^A^	89.66 ± 0.46^A^	90.04 ± 0.46^A^	0.767^NS^
	3	61.70 ± 0.71^bB^	65.16 ± 0.74^aB^	67.16 ± 0.72^aB^	0.000^**^
	5	40.54 ± 1.11^bC^	44.95 ± 1.47^aC^	47.62 ± 1.38^aC^	0.001^**^
	*P*-value	0.000^**^	0.000^**^	0.000^**^	–
Plasma membrane integrity (%)	0	63.75 ± 1.24^A^	64.29 ± 1.39^A^	65.00 ± 1.42^A^	0.808^NS^
	3	30.58 ± 0.58^cB^	33.00 ± 0.55^bB^	36.12 ± 0.50^aB^	0.000^**^
	5	10.95 ± 0.58^cC^	14.00 ± 0.37^bC^	17.25 ± 0.44^aC^	0.000^**^
	*P*-value	0.000^**^	0.000^**^	0.000^**^	–
Acrosomal integrity (%)	0	91.20 ± 0.43^A^	91.70 ± 0.53^A^	91.79 ± 0.71^A^	0.741^NS^
	3	70.12 ± 0.64^cB^	73.04 ± 0.73^bB^	75.54 ± 0.67^aB^	0.000^**^
	5	48.45 ± 1.10^cC^	51.70 ± 0.94^bC^	54.91 ± 0.96^aC^	0.000^**^
	*P*-value	0.000^**^	0.000^**^	0.000^**^	–
MMP (%)	0	86.33 ± 0.45^A^	86.54 ± 0.73^A^	87.00 ± 1.00^A^	0.821^NS^
	3	58.00 ± 0.50^cB^	60.87 ± 0.59^bB^	64.16 ± 0.52^aB^	0.000^**^
	5	35.41 ± 0.69^cC^	37.62 ± 0.71^bC^	41.79 ± 0.79^aC^	0.000^**^
	*P*-value	0.000^**^	0.000^**^	0.000^**^	–

^**^p < 0.01; ^NS^Non-significant.

Means with different superscripts in a row (a, b, c) differed significantly.

Means with different superscripts in a column (A, B, C) differed significantly.

At day 0, sperm viability was comparable among treatments (89.62 ± 0.40%−90.04 ± 0.46%). Viability declined significantly (*P* < 0.01) during storage in all groups; however, AuNP-treated semen maintained higher viability than the control. By day 5, viability values were 40.54 ± 1.11%, 44.95 ± 1.47%, and 47.62 ± 1.38% in T0, T1, and T2, respectively.

A similar pattern was observed for plasma membrane integrity. Initial values ranged from 63.75 ± 1.24% to 65.00 ± 1.42%, while day-5 values declined to 10.95 ± 0.58%, 14.00 ± 0.37%, and 17.25 ± 0.44% in T0, T1, and T2, *respectively (P*<*0.01)*.

Acrosomal integrity was also better preserved in AuNP-treated groups. On day 5, acrosomal integrity was highest in T2 (54.91 ± 0.96%), followed by T1 (51.70 ± 0.94%) and T0 (48.45 ± 1.10%) (*P* < 0.01).

Mitochondrial membrane potential assessed by JC-1 staining declined significantly during storage but remained higher in AuNP-supplemented treatments. The proportion of spermatozoa exhibiting high MMP on day 5 was 35.41 ± 0.69%, 37.62 ± 0.71%, and 41.79 ± 0.79% in T0, T1, and T2, respectively (*P* < 0.01). Fluorescence microscopic observations from JC-1, CFDA/PI, and FITC-PNA staining supported these quantitative findings by showing a greater proportion of viable, membrane-intact, and acrosome-intact spermatozoa in AuNP-treated semen samples.

### 3.3 Effect of AuNP supplementation on oxidative stress and antioxidant status

The oxidative stress indices of boar semen during liquid storage are presented in Table 3. Malondialdehyde (MDA) concentration increased significantly (*P* < 0.01) with storage duration in all treatment groups. However, AuNP supplementation reduced MDA accumulation compared with the control. On day 5, MDA concentrations were 2.29 ± 0.06, 2.03 ± 0.04, and 1.80 ± 0.05 μmol/ml in T0, T1, and T2, respectively.

**Table 3:** Effect of different doses of AuNPs supplementation on semen extender in various oxidative stress parameters.

Parameters	Day	T0	T1	T2	*P*-value
MDA (μmol/ml)	0	0.94 ± 0.01^aC^	0.92 ± 0.01^abC^	0.89 ± 0.01^bC^	0.009^**^
	3	1.69 ± 0.06^aB^	1.56 ± 0.05^aB^	1.39 ± 0.04^bB^	0.001^**^
	5	2.29 ± 0.06^aA^	2.03 ± 0.04^bA^	1.80 ± 0.05^cA^	0.000^**^
	*P*-value	0.000^**^	0.000^**^	0.000^**^	–
TAC (nmol/mL)	0	901.66 ± 8.33^A^	908.33 ± 7.26^A^	912.08 ± 9.04^A^	0.666^NS^
	3	656.25 ± 10.83^cB^	692.29 ± 11.46^bB^	747.91 ± 14.56^aB^	0.000^**^
	5	536.66 ± 10.22^cC^	578.33 ± 8.83^bC^	607.50 ± 8.63^aC^	0.000^**^
	*P*-value	0.000^**^	0.000^**^	0.000^**^	–

^**^p < 0.01; ^NS^Non-significant. Means with different superscripts in a row (a, b, c) differed significantly.

Means with different superscripts in a column (A, B, C) differed significantly.

Total antioxidant capacity (TAC) declined during storage irrespective of treatment. Nevertheless, TAC remained significantly higher in AuNP-supplemented semen than in the control group. On day 5, TAC values were 536.66 ± 10.22, 578.33 ± 8.83, and 607.50 ± 8.63 nmol/ml in T0, T1, and T2, respectively (*P* < 0.01).

## 4 Discussion

The present study demonstrated that supplementation of BTS extender with gold nanoparticles (AuNPs) significantly improved the preservation of boar spermatozoa during liquid storage at 17 °C. Although all semen quality attributes deteriorated progressively with increasing storage duration, the decline was considerably slower in AuNP-supplemented groups, particularly at 10 million AuNPs/ml (T2). The beneficial effects of AuNPs were evident in sperm motility, membrane integrity, acrosomal status, mitochondrial activity, and antioxidant defense mechanisms, suggesting that AuNPs effectively mitigate storage-induced oxidative and structural damage in boar spermatozoa.

Sperm motility is one of the most important indicators of fertilizing potential and is highly dependent on the integrity of the plasma membrane, mitochondrial function, and intracellular ATP production. In the present study, AuNP supplementation significantly improved total and progressive motility as well as velocity parameters (VCL, VAP, and VSL) throughout the storage period. The superior preservation of motion characteristics observed in T2 indicates that AuNPs may protect the sperm flagellar apparatus and mitochondrial energy-generating systems from oxidative deterioration. During liquid storage, boar spermatozoa are particularly susceptible to oxidative damage because their plasma membranes contain high concentrations of polyunsaturated fatty acids and relatively low levels of endogenous antioxidant enzymes (21). Reactive oxygen species (ROS) generated during storage induce lipid peroxidation, resulting in loss of membrane fluidity, impaired ion transport, reduced ATP synthesis, and consequently diminished sperm motility. Gold nanoparticles have been reported to possess strong free-radical scavenging properties and can neutralize superoxide anions, hydroxyl radicals, and hydrogen peroxide, thereby preserving cellular function (22).

The improved motility observed in the present study agrees with the findings of Ismail et al. (12), who reported enhanced post-thaw motility and viability in goat spermatozoa supplemented with AuNPs. Similarly, Khalil et al. (11) observed improved kinetic parameters and fertilizing capacity in buffalo semen following AuNP supplementation. Comparable protective effects of nanoparticles have also been reported with selenium nanoparticles in boar semen (3) and zinc oxide nanoparticles in ram semen (23), indicating that nanoparticle-based antioxidant systems may represent an emerging strategy for semen preservation.

Apart from motility, AuNP supplementation significantly enhanced sperm viability, plasma membrane integrity, and acrosomal integrity during storage. These structures are highly vulnerable to oxidative injury because ROS-mediated lipid peroxidation causes disruption of phospholipid bilayers, increased membrane permeability, and leakage of intracellular enzymes (24). Preservation of membrane integrity is essential for maintaining osmotic balance, ion exchange, capacitation processes, and fertilization competence. The higher proportion of viable and membrane-intact spermatozoa observed in the AuNP-treated groups suggests that AuNPs exert a membrane-stabilizing effect during storage. The fluorescence images obtained using CFDA/PI and FITC-PNA staining further confirmed the superior preservation of sperm structural integrity in AuNP-supplemented semen.

The improved acrosomal integrity observed in the present study is of particular importance because the acrosome contains hydrolytic enzymes required for penetration of the zona pellucida during fertilization. Damage to the acrosomal membrane during storage reduces the fertilizing ability of spermatozoa even when motility is retained. The higher percentage of acrosome-intact spermatozoa in AuNP-treated groups suggests that AuNPs protect both outer and inner acrosomal membranes from oxidative and cold-shock-induced damage. Similar observations have been reported in cryopreserved goat semen treated with AuNPs (12) and in boar semen supplemented with antioxidant compounds such as melatonin and resveratrol (25, 26).

Mitochondrial membrane potential (MMP) is considered a reliable indicator of sperm metabolic activity and fertilizing capacity because ATP generated through oxidative phosphorylation is required for sperm motility and survival. In the present study, AuNP supplementation significantly preserved MMP throughout storage, as demonstrated by JC-1 staining. The higher proportion of spermatozoa exhibiting high mitochondrial membrane potential in T2 suggests that AuNPs protect mitochondrial membranes from oxidative injury and maintain efficient electron transport chain activity. Mitochondria are both a major source and target of ROS in spermatozoa; excessive ROS production disrupts mitochondrial membrane integrity, leading to reduced ATP synthesis and initiation of apoptotic pathways (27). By limiting oxidative stress, AuNPs may preserve mitochondrial function and sustain energy production during prolonged storage. Similar improvements in mitochondrial activity have been reported following supplementation of boar semen with antioxidants such as selenium nanoparticles and oxyrase (3, 28).

The most notable finding of the present study was the substantial reduction in oxidative stress markers in AuNP-treated semen. Lipid peroxidation, measured by malondialdehyde (MDA) concentration, increased progressively during storage in all treatment groups; however, the increase was significantly lower in semen supplemented with AuNPs. Conversely, total antioxidant capacity remained significantly higher in AuNP-treated groups throughout storage. These findings indicate that AuNPs effectively enhance the antioxidant defense system of spermatozoa and reduce ROS-mediated cellular damage.

Boar spermatozoa are particularly vulnerable to oxidative stress because of their high content of docosahexaenoic and arachidonic acids in the plasma membrane (5). Excessive ROS generation during storage initiates chain reactions of lipid peroxidation that compromise membrane integrity, mitochondrial function, and DNA stability. The lower MDA concentrations and higher TAC values observed in the present study indicate that AuNPs may interrupt these oxidative chain reactions and preserve cellular homeostasis. Similar reductions in lipid peroxidation following AuNP supplementation have been reported in goat semen (12), buffalo semen (11), and other mammalian cell systems (29). Furthermore, antioxidant-mediated improvements in boar semen quality have been widely reported following supplementation with vitamin E, glutathione, melatonin, sericin, selenium nanoparticles, and oxyrase (3, 25, 28), supporting the central role of oxidative stress in determining semen storage longevity. Additionally, because gold nanoparticles have antibacterial and antifungal qualities that prevent the growth of dangerous germs (30), they can help preserve semen by lowering contamination and preserving semen quality while it is being stored. Future studies involving a larger number of ejaculates or pooling ejaculates from multiple boars before semen processing would help minimize the influence of individual boar variation and further validate the observed treatment effects.

## 5 Conclusions

In conclusion, supplementation of gold nanoparticles (AuNPs) in boar semen extender improved the preservation of sperm quality during liquid storage by mitigating oxidative stress and maintaining cellular integrity. Among the concentrations tested, 10 million AuNPs/ml was the most effective, resulting in superior sperm motility, viability, membrane integrity, mitochondrial function, and reduced lipid peroxidation throughout the 5-day storage period. The observed benefits are likely associated with the antioxidant properties of AuNPs, which enhance cellular defense mechanisms and protect spermatozoa from storage-induced damage. These findings suggest that AuNP supplementation may serve as a promising strategy for extending the shelf life of liquid-preserved boar semen and improving the efficiency of artificial insemination programs. Further studies are warranted to elucidate the underlying molecular mechanisms, assess long-term biosafety, and validate fertility outcomes under commercial field conditions.

## Data Availability

The original contributions presented in the study are included in the article/supplementary material, further inquiries can be directed to the corresponding author.
